# A Prehepatectomy Circulating Exosomal microRNA Signature Predicts the Prognosis and Adjuvant Chemotherapeutic Benefits in Colorectal Liver Metastasis

**DOI:** 10.3390/cancers13174258

**Published:** 2021-08-24

**Authors:** Yun Wang, Xiuxing Chen, Haocheng Lin, Xiaoqiang Sun, William Pat Fong, Xiaojun Wu, Zhizhong Pan, Yunfei Yuan, Jieying Liang, Deshen Wang, Ziming Du, Baocai Xing, Yuhong Li

**Affiliations:** 1 Department of Hematologic Oncology, State Key Laboratory of Oncology in South China, Collaborative Innovation Center for Cancer Medicine, Sun Yat-Sen University Cancer Center, Guangzhou 510060, China; wangyun@sysucc.org.cn; 2 Department of Medical Oncology, State Key Laboratory of Oncology in South China, Collaborative Innovation Center for Cancer Medicine, Sun Yat-Sen University Cancer Center, Guangzhou 510060, China; chenxx43@mail2.sysu.edu.cn (X.C.); linhch@sysucc.org.cn (H.L.); will3_pf@hotmail.com (W.P.F.); liangjy@sysucc.org.cn (J.L.); wangdsh@sysucc.org.cn (D.W.); 3 Department of Oncology, Sun Yat-Sen Memorial Hospital of Sun Yat-Sen University, Guangzhou 510120, China; 4 Department of Medical Oncology, Guangzhou Concord Cancer, Guangzhou 510060, China; 5 School of Mathematics, Sun Yat-Sen University, Guangzhou 510275, China; sunxq6@mail.sysu.edu.cn; 6 Department of Colorectal Surgery, State Key Laboratory of Oncology in South China, Collaborative Innovation Center for Cancer Medicine, Sun Yat-Sen University Cancer Center, Guangzhou 510060, China; wuxj@sysucc.org.cn (X.W.); panzhzh@sysucc.org.cn (Z.P.); 7 Department of Hepatobiliary Surgery, State Key Laboratory of Oncology in South China, Collaborative Innovation Center for Cancer Medicine, Sun Yat-Sen University Cancer Center, Guangzhou 510060, China; yuanyf@sysucc.org.cn; 8 Department of Molecular Diagnostics, State Key Laboratory of Oncology in South China, Collaborative Innovation Center for Cancer Medicine, Guangdong Key Laboratory of Nasopharyngeal Carcinoma Diagnosis and Therapy, Sun Yat-Sen University Cancer Center, Guangzhou 510060, China; duzm1@sysucc.org.cn; 9 Hepatopancreatobiliary Surgery Department I, Key Laboratory of Carcinogenesis and Translational Research, Ministry of Education, Peking University School of Oncology, Beijing Cancer Hospital and Institute, Beijing 100143, China

**Keywords:** exosomal miRNA, liver metastasis, hepatectomy, colorectal cancer, prognosis

## Abstract

**Simple Summary:**

Exosomal miRNAs are associated with colorectal cancer liver metastasis (CRLM)-related biological behavior and prognosis. However, an exosomal miRNA signature predicting postoperative survival and the value of adjuvant chemotherapy for CRLM remains elusive. Using miRNA sequencing and the LASSO model, we constructed an miRNA signature comprising four exosomes. The signature showed a good predictive performance for patient outcome and the advantage of adjuvant chemotherapy after hepatectomy in two institutions’ training and validation cohorts. In addition, we found that the four miRNAs could target signaling molecules playing crucial roles in colorectal cancer metastasis, vesicle-related processing, and T cell activation. Furthermore, the exosomal miRNA score also increased with the decreasing Immunoscore. We believe that our signature can predict the prognosis and guide adjuvant chemotherapy decisions after liver metastasectomy in CRLM patients, further improving the predictive performance of the current CRLM predictive model system.

**Abstract:**

Background: The clinical risk score (CRS) for prediction and treatment decision in colorectal liver metastasis (CRLM) is important, but imprecise. Exosomal miRNAs play critical roles in CRLM-related biological behavior. However, an exosomal miRNA score system for predicting posthepatectomy survival and the adjuvant chemotherapy benefit of CRLM remains elusive. Methods: miRNA sequencing was used to identify differentially expressed miRNAs, and the LASSO model was used to select miRNAs to construct the intent model. The predictive performance of the model was evaluated by the area under the ROC curve (AUC) in the training, internal validation, and external validation cohorts. Results: Sixteen differentially expressed exosomal miRNAs were identified, and four miRNAs were selected for model construction. Our model performed well in predicting prognosis with five-year AUCs of 0.70 (95% CI: 0.59–0.81), 0.70 (0.61–0.81), and 0.72 (057–0.86) in the training, internal, and external validation cohorts, respectively. miRNA classifier high-risk patients had better survival benefit from adjuvant chemotherapy regardless of CRS. All four miRNAs target signaling molecules play crucial roles in colorectal cancer metastasis, vesicle-related processing, and T cell activation. It also negatively correlated with the liver metastasis Immunoscore. Conclusion: We developed a circulating exosomal miRNA signature that can predict the prognosis and guide adjuvant chemotherapy decisions after hepatectomy in CRLM.

## 1. Introduction

Colorectal cancer (CRC) is the third most common malignancy and the second leading cause of cancer-related death [1]. The liver is the most common site of distant metastasis [2,3]. Despite the advances in hepatectomy and adjuvant therapies, the five-year survival rate of colorectal cancer liver metastases (CRLM) remains only 25–50% [4]. The clinical risk score (CRS) is the most widely used predictive system for postoperative outcomes and treatment guides in CRLM [5]. Nevertheless, the survival of patients with the same CRS may vary considerably, indicating that a better predictive biomarker is needed in the era of precision medicine [6,7].

Exosomes are extracellular vesicles of 30–150 nm in diameter, which carry molecular information derived from various cell types [8]. Emerging evidence has shown that exosomes are fundamental mediators in the CRLM microenvironment and are critical for cell signaling, immune response, and metabolism regulation of CRLM development [9]. Circulating exosomal miRNAs contain a plethora of bio information, making them ideal biomarkers for disease monitoring and prognosis [10]. Several studies found that circulating miRNAs could predict prognosis and metastasis in CRC [11,12]. Moreover, serum exosomal miR-122 is a potential diagnostic and prognostic biomarker of CRLM [13]. Cancer-derived exosomal miR-25-3p promotes CRLM by inducing vascular permeability and angiogenesis [14]. Hence, the biological importance of exosomal miRNAs in CRLM cannot be understated.

Based on this, we intend to establish a CRLM-related circulating exosomal miRNA signature to predict the posthepatectomy prognosis and guide adjuvant chemotherapy decisions in CRLM.

## 2. Materials and Methods

### 2.1. Study Design and Patients

Six paired plasma samples from nonmetastatic CRC and CRLM patients were matched by sex, age, primary tumor sites, T-stage, and N-stage. The plasma exosomal miRNAs were extracted and sequenced for the differential exosomal miRNAs by the Illumina Hiseq 2500/2000 platform. After quality control and clean tags filtering, the miRNA expression profiles were aligned and identified according to the miRBase database in Release 21. The differentially expressed miRNA were revalidated in another 10 nonmetastatic CRC and 20 CRLM plasma samples by qRT-PCR.

We further assessed the prognostic value of the candidate CRLM-associated exosomal miRNA. Exosomal miRNAs were extracted from 395 CRLM patients. Patients who underwent liver metastasectomy with curative intent and had adequate prehepatectomy serum samples and clinicopathological information were included. Patients with metastases in sites other than the liver or a history of prior hepatectomy, insufficient exosomal miRNA extraction, and substandard miRNA specimens were excluded. For the training and internal validation set, data were collected from 227 patients from the Sun Yat-sen University Cancer Center (SYSCC) between May 2002 and September 2015. Computer-generated random numbers were used to assign 113 of these patients for the training set and 114 for the internal validation set. Another 168 patients from the Peking University Cancer Center, between May 2011 and December 2016, were recruited as an independent external validation set. The study was approved by the institutional ethical review boards of the included hospitals.

### 2.2. Extraction and Quantification of Exosomal miRNAs

Plasma and serum exosomes were isolated using the Invitrogen™ Total Exosome Isolation Kit (Invitrogen, CA, USA) and ExoQuick™ exosome precipitation solution (System Biosciences, CA, USA), respectively. The extracted exosomes were examined by the JEM-1400 electron microscope (JEOL, Tokyo, Japan), NanoSight NS300 (Malvern, Cambridge, UK), and Western blot analysis with markers as previously reported [15]. Small RNAs were extracted from the exosomes using the NucleoZOL reagent (Macherey-Nagel, Duren, Germany). Commercial external control for miRNA (TIANGEN BIOTECH, Beijing, China) was used as a reference and detection by the corresponding primer. Exosomal miRNAs were reverse transcribed and detected by qRT-PCR using the All-in-one™ miRNA qRT-PCR Kit (GeneCopoeia, Maryland, VA, USA). The relative expression of exosomal miRNA was estimated by the 2−ΔΔCt method (ΔCt = CtmiRNA-Ctexternal control).

### 2.3. Circulating Exosomal microRNA Signature Construction and Validation

The expression of exosomal miRNAs was detected and grouped into high and low levels based on the optimal cutoff value in the training cohort. High values of miRNA expression were scored as 1, while low values were scored as 0. The circulating exosomal miRNA signature was identified using the expression level of miRNAs by the least absolute shrinkage and selection operator (LASSO) Cox regression analysis. The robustness of the exosomal miRNA signature was validated in the internal validation and external validation cohorts.

### 2.4. Origin Identification of Circulating Exosomal miRNAs

To identify the origin of the model-related exosomal miRNA, we isolated tumor cells from fresh CRLM-resected specimens and corresponding monocytes, T lymphocytes, B lymphocytes, and natural killer (NK) cells from the peripheral blood of five CRLM patients as previously reported [16]. Cells were seeded at a density of 1 × 106 cells/mL for 48 h. The exosomes were isolated from the supernatants of the cultured tumor cells and immune cells using Total Exosome Isolation Reagent (from cell culture media) (Invitrogen, CA, USA).

### 2.5. Statistical Analysis

Category variables were compared with the χ2 test or Fisher’s exact test. The collinearity of exosomal miRNA levels was examined by Spearman’s correlation test. Overall survival (OS) and relapse-free survival (RFS) were calculated from the date of liver metastasectomy to death due to any cause and the first relapse or death, respectively. Kaplan–Meier curves with the log-rank test were generated using the “survival” package. The prognostic performances of clinicopathological variables were explored via univariate and multivariate Cox regression analyses. The optimal cutoff values of the circulating miRNA expression and the exosomal miRNA signature were determined by the “survminer” package with a minprop (the minimal proportion of the observations per group) of 30% in the training cohort. Time-dependent receiver operating characteristic (ROC) curves were used to assess the predictive performance of the circulating exosomal miRNA signature by the survival ROC package. The confidence intervals were assessed by the bootstrap method. A nomogram was used to integrate the CRS and exosomal miRNA signature with the assessment of consistency by calibration. All statistical analyses were performed using the R software (Version 3.6.0) and the SPSS Version 24.0 software (SPSS, Chicago, IL, USA). A two-sided *p*-value < 0.05 was considered statistically significant.

## 3. Results

### 3.1. Identification of Differentially Expressed Exosomal miRNAs

**Figure 1:**
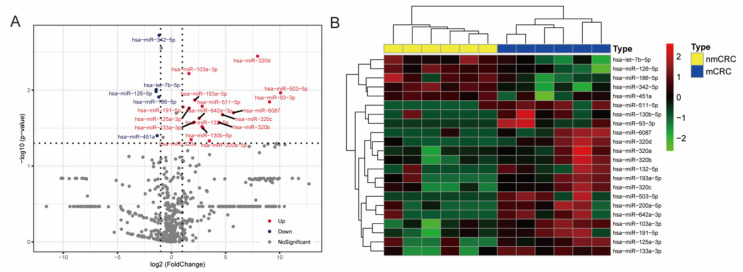
Differential expression of circulating exosomal miRNA among 6 paired nonmetastatic CRC and CRLM plasma specimens. (**A**) Volcano plot of the identified differentially expressed circulating exosomal miRNAs, with fold change > 2.0 and *p*-value < 0.05 considered significant; in the volcano plot, upexpression and downexpression miRNAs are colored in red and blue, respectively. (**B**) Hierarchical clustering of the 22 differential circulating exosomal miRNA expression from 6 patients with nonmetastatic CRC (in yellow) and 6 CRLM patients (in blue). Each row represents an individual miRNA, and each column represents an individual sample. The color shade indicates relative expression levels from low to high on a log2 scale from −2.0 to 2.0. CRC, colorectal cancer; CRLM, colorectal cancer liver metastases.

The study was carried out as described in the flowchart (Figure S1). The isolated exosomes were identified by electron microscopy, NanoSight NS300, and Western blot (Figure S2). The RNA sequencing analysis of six paired nonmetastatic CRC and CRLM plasma specimens identified 22 differentially expressed circulating exosomal miRNAs according to the criteria of fold change > 2.0 and *p*-value < 0.05. Among them, eighteen miRNAs were upregulated, while four were downregulated (Figure 1A,B). The twenty-two differentially expressed miRNAs were then divided into two discrete groups by hierarchical clustering (Figure 1B). An independent qRT-PCR analysis of 10 nonmetastatic CRC and 20 CRLM plasma samples further confirmed that 16 out of the 18 upregulated exosomal miRNAs were differentially expressed (Figure S3).

### 3.2. Construction and Validation of a Circulating Exosomal miRNA Signature

**Figure 2:**
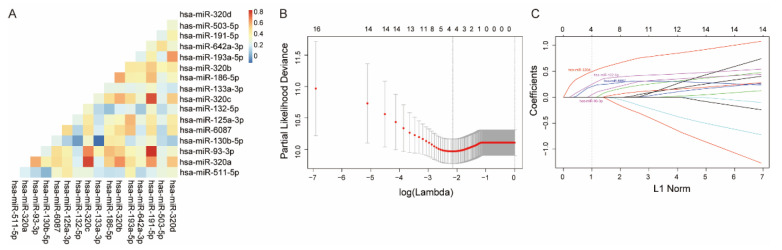
Construction of CRLM circulating exosomal miRNA models. (**A**) The correlation heatmap shows the collinearity of 16 candidate miRNAs in the training cohort. The color in each cell represents the correlation coefficient of the Spearman correlation between the row and column corresponding exosomal miRNA expression level (binary variable). (**B**) The optimal weighting coefficient of the exosomal miRNA model was determined by a least absolute shrinkage and selection operator (LASSO) Cox regression model. A 1000-fold cross-validation was applied to select the tuning parameters based on the partial likelihood deviance of the different numbers of variables. The optimal values by the minimum criteria and 1-SE criteria are emphasized with dotted vertical lines. (**C**) The LASSO coefficient profiles of the circulating exosomal miRNA are drawn in the training cohort. Each curve corresponds to a candidate circulating exosomal miRNA. The vertical line was drawn at the optimal *λ* value determined by the minimum criteria. CRLM, colorectal cancer liver metastases.

We further collected 395 serum specimens from CRLM patients from two independent institutions (Figure S1). Table 1 shows the clinicopathological characteristics of the patients. The median follow up was 50.6 months for patients from SYSUCC and 32.7 months for those from Peking University Cancer Center. The expressions of 16 circulating miRNA were examined in the training cohort and separated into high and low levels as mentioned in the Methods. Collinearity was observed among the expression levels of miRNAs (Figure 2A). The LASSO Cox regression model was applied to identify four circulating exosomal miRNAs with optimal weighting coefficients in the training cohort (Figure 2B,C). A formula was generated to calculate the exosomal miRNA risk score for the risk of death after hepatectomy for each patient, where: risk score = (0.198 × levels of miR-6087) + (0.236 × levels of miR-132-5p) + (0·034 × levels of miR-93-3p) + (0.484 × levels of miR-320d). The four exosomal miRNAs also showed a significant prognostic role for overall survival (OS) in the training cohort (Figure S3).

**Figure 3:**
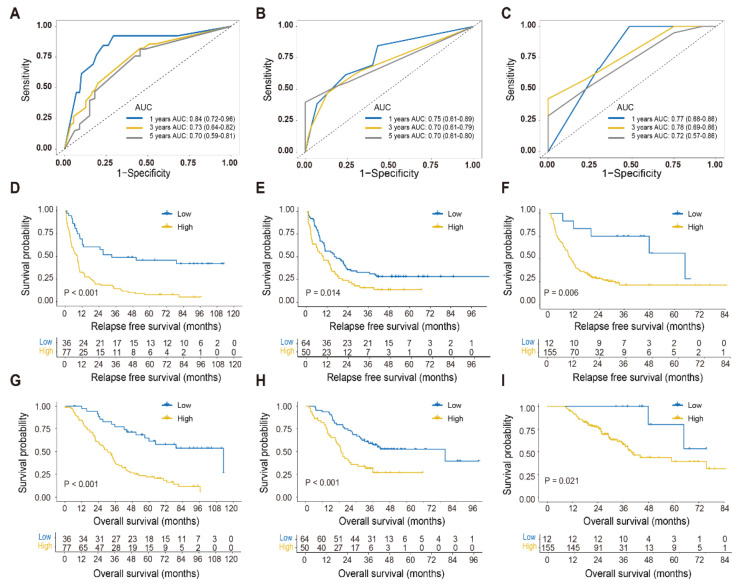
Time-dependent receiver operating characteristic (ROC) curves and Kaplan–Meier curves of relapse-free survival and overall survival for the circulating exosomal miRNA model. (**A**–**C**) Time-dependent ROC curves based on the circulating exosomal miRNA score in the training (**A**), internal validation (**B**), and external validation cohort (**C**). The prognostic accuracy of the model was assessed by the 1-, 3-, and 5-year AUCs of overall survival. The 95% CI was estimated by the bootstrap method. (**D**–**I**) Kaplan–Meier curves for relapse-free survival and overall survival in different exosomal miRNA risk CRLM groups in the training (**D**,**G**), internal validation (**E**,**H**), and external validation cohorts (**F**,**I**). CRLM, colorectal cancer liver metastases; AUC, area under the curve; CI, confidence interval.

The AUC values of the circulating exosomal miRNA signature for 1-, 3-, and 5-year survival were 0.84, 0.73, and 0.70 in the training cohort (Figure 3A). The parallel values were 0.75, 0.70, and 0.70 in the internal validation cohort (Figure 3B) and 0.77, 0.78, and 0.72 in the external validation cohort (Figure 3C), respectively. Based on the consensus optimal cutoff determined in the training cohort, the exosomal miRNA signature was further sorted into high and low exosomal miRNA risk statuses in each cohort. Patients with higher exosomal miRNA risk had impaired the 3-year RFS survival rates of in the training cohort (14.3% vs. 48.9%, *p* < 0.001), internal validation cohort (16.0% vs. 31.3%, *p* = 0.014), and external validation cohort (21.2% vs. 75.0%, *p* = 0.006) (Figure 3D–F). Consistently, patients with low exosomal miRNA risk had better five-year OS rates in the training cohort (61.5% vs. 25.1%, *p* < 0.001), internal validation cohort (52.7% vs. 27%, *p* < 0.001), and external validation cohort (80.0% vs. 58.0%, *p* = 0.021) (Figure 3G–I). The univariate Cox regression indicated the prognostic value of tumor differential grade, N-stage, and CRS-related factors in RFS and OS (Table 2). The prognostic role of ablation may be biased by the imbalance of the liver metastasis burden. After adjustment for other clinicopathological factors, the four-exosomal miRNA signature remained a significant and independent factor for RFS and OS in the three cohorts (Table S1).

### 3.3. Merged Score Based on the CRS and Exosomal miRNA Signature

**Figure 4:**
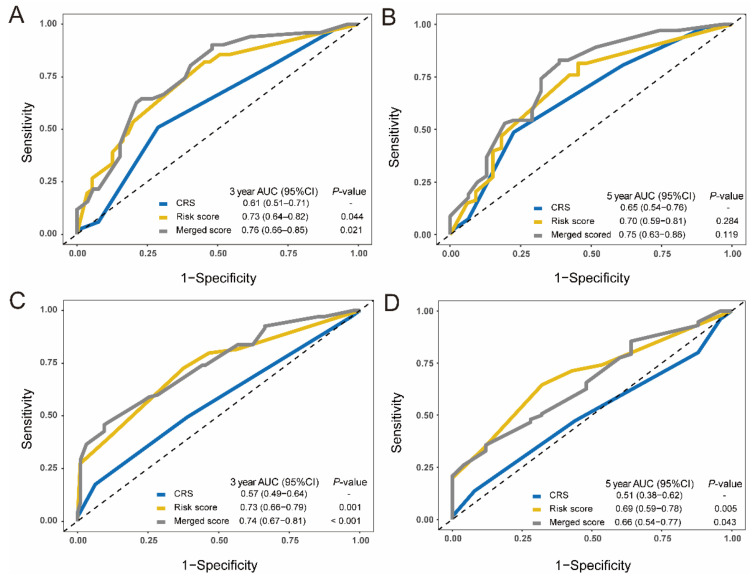
Comparisons of the prognostic accuracy among the CRS system, exosomal miRNA score, and merged score. (**A**,**B**). Time-dependent ROC curves are drawn in the training cohorts. The 3-year AUCs (**A**) and 5-year AUCs (**B**) of overall survival were calculated for the CRS, 4-exosomal miRNA risk score, and a merged score that was constructed by the CRS and exosomal miRNA risk score. (**C**,**D**) Time-dependent ROC curves of the CRS, exosomal miRNA risk score and the merged score in the combined validation cohorts. The 3-year AUCs (**C**) and 5-year AUCs (**D**) of overall survival were evaluated for the three score systems. The AUC of the CRS system was identified as the control. The two-sided *p*-values were calculated through the bootstrap test. ROC, receiver operating characteristic; AUC, area under the curve; CRS, clinical risk score.

The CRS and exosomal miRNA signature were merged into a more sensitive predictive system by nomogram. The predictive accuracy was examined by calibration plots in the training and validation cohorts (Figure S4A–C). The AUC of the merged score was significantly larger than the classic CRS model for the three-year OS prediction (0.76 vs. 0.61, *p* = 0.021), but was numerically larger for five-year OS prediction in the training cohort (0.75 vs. 0.65, *p* = 0.119) (Figure 4A,B). The cumulative advantage of OS prediction for the merged score was also observed in the combined validation cohort (Figure 4C,D).

### 3.4. Candidate Factors Identifying the Benefit of Adjuvant Chemotherapy

**Figure 5:**
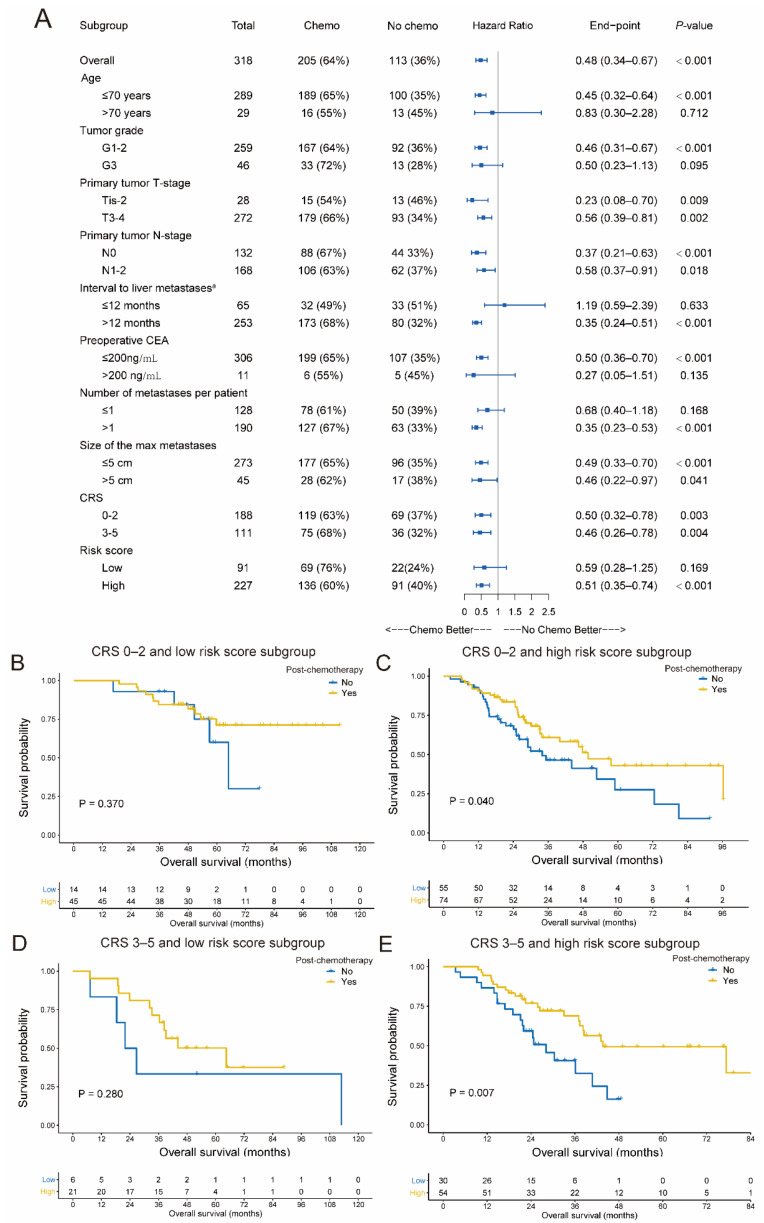
Subgroup analysis for the postoperative chemotherapy advantage. (**A**) The forest plot for the survival advantage for postoperative chemotherapy after CRLM resection was stratified by several clinicopathological features based on the Cox models. The HR and 95% CIs are visually presented with blue squares and error bars. (**B**–**D**) The postoperative chemotherapy survival advantage was assessed in four subgroups stratified by both the CRS system and 4-exosomal miRNA risk score. The Kaplan–Meier curves for overall survival for with or without postsurgery chemotherapy in CRS 0–2 and low exosomal miRNA risk subgroups (**B**), CRS 0–2 and high exosomal miRNA risk subgroups (**C**), CRS 3–5 and low exosomal miRNA risk subgroups (**D**), and CRS 3–5 and high exosomal miRNA risk subgroups (**E**), respectively. CRLM, colorectal cancer liver metastases; HR, hazards ratio; CIs, confidence intervals; CRS, clinical risk score.

To explore the potential predictive factors for the benefit post chemotherapy, subgroup analysis was performed in patients stratified by candidate variables. Ages ≤ 70 years, G1–2 tumor grade, interval from primary tumor surgery to liver metastases > 12 months, preoperative carcinoembryonic antigen (CEA) ≤ 200 ng/mL, more than one liver lesion, and high exosomal miRNA risk were the benefit factors for adjuvant chemotherapy (Figure 5A). Additionally, patients with the miRNA-classifier-defined high risk had a survival advantage in adjuvant chemotherapy regardless of CRS status, while low-risk patients showed no advantage (Figure 5B–E).

### 3.5. Origin and Mechanism of the Model-Included miRNAs

To identify whether the intent miRNAs were properly detected by qPCR, we connected the qPCR product to a plasmid template for Sanger sequencing. The results showed that all four intent miRNAs could be correctly detected (Figure S5).

**Figure 6:**
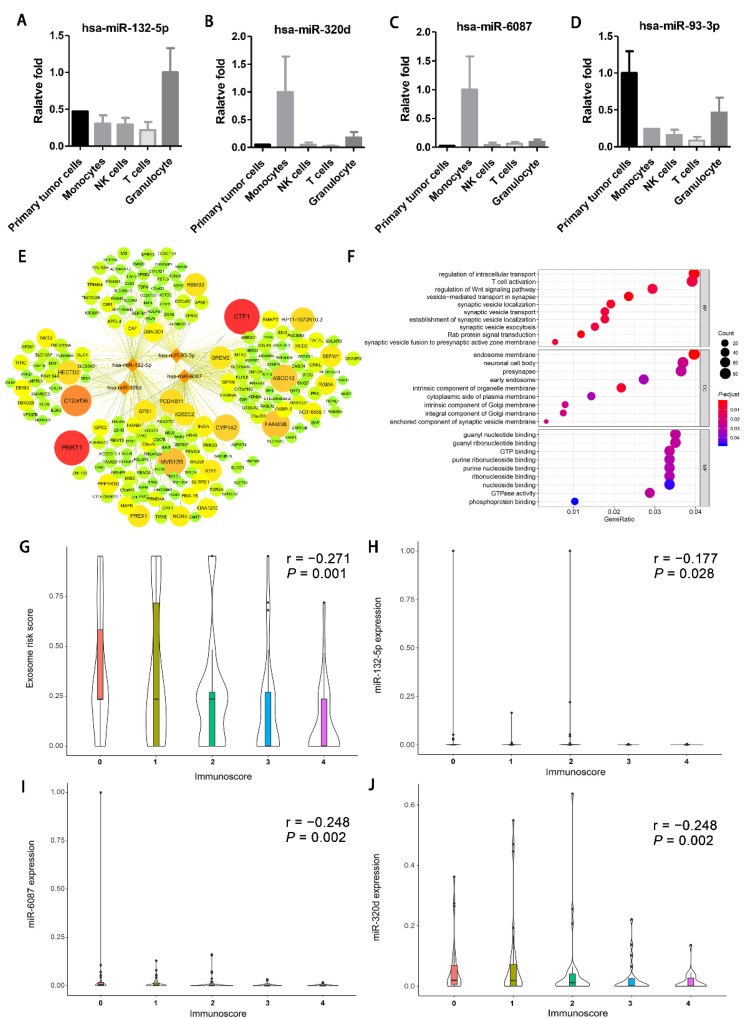
The prediction mechanism and origins of the model-included miRNAs. (**A**–**D**) The histogram of the origins of the 4 model-included exosomal miRNAs, has-miR-132-5p (**A**), has-miR-320d (**B**), has-miR-6087 (**C**), and has-miR-93-3p (**D**); qPCR was performed to calculate the expression of the 4 miRNAs in the exosomes isolated from the exosome-free supernatant of the cultured tumor cells, monocytes, NK cells, T cells, and granulocytes (mean ± SEM). (**E**) miRNA-mRNA regulatory network of the 4 model-included miRNAs and the target genes predicted by the TARGETSCAN website. The relationship between the miRNA and mRNA was assessed by cumulative weight context++ core and visually presented by the color of the line and the size of the target mRNA. Only the targeted genes with the absolute value of the assessed cumulative weight context^++^ core less than 0.2 and targeted by more than two miRNAs are presented. (**F**) GO plot of the target genes of the 4-model miRNA. Bubble map of the top 10 pathway terms derived from functional enrichment of each GO subset (biological processes (BP), cell component (CC) and molecular function (MF)). Each bubble indicates an enrichment. The size and color of the bubble indicates the degree of the strength and significance of the enrichment. (**H**–**J**) The correlation between the Immunoscore and the circulating exosomal miRNA risk score (**G**), the expression of exosomal miR-132-5p (**H**), miR-6087 (**I**), and miR-320d (**J**) based on Spearman correlation analysis. The correlation between the Immunoscore and the expression of exosomal miR-93-3p is not shown due to the lack of statistical significance. SEM, standard error of the mean.

Exosomal miRNAs from the CRLM tumor cells and the corresponding autologous immune cells were extracted and examined. miR-132-5p was high in the exosomes of granulocytes (Figure 6A). miR-320d and miR-6087 were preferentially expressed in the exosomes of monocytes (Figure 6B,C). Furthermore, both primary tumor cells and granulocyte exosomes showed a high expression of miR-93-3p (Figure 6D). Potential target genes of the model-included miRNAs were also explored by TargetScan (Figure 6E). Gene ontology (GO) analysis of the candidate target genes showed enrichment mainly in vesicle-related processing, T cell activation, and the Wnt signaling pathway (Figure 6F).

To further investigate the correlation between immune regulation and the 4-miRNA signature, the Immunoscore data of 173 of the 273 patients in SYSUCC were extracted and analyzed [17]. The Immunoscore increased with decreasing exosomal miRNA score and decreasing expression of exosomal miRNA132-5p, miRNA6087, and miRNA320d (Figure 6G–J).

## 4. Discussion

Circulating exosomal miRNAs have added advantages as predictive biomarkers because they can be noninvasively analyzed before surgery. In this study, we brought forward an exosomal miRNA signature based on the levels of circulating exosomal miR-6087, miR-132-5p, miR-93-3p, and miR-320d, which showed good performance in predicting CRLM postoperative survival. Furthermore, the merged score of the CRS and our exosomal miRNA signature showed a better three-year survival predictive value than each predictive system independently. Patients with a high risk had survival advantages with adjuvant chemotherapy regardless of the CRS. Our results suggest that this exosomal miRNA signature can potentially predict the prognosis and guide adjuvant chemotherapy decision for CRLM patients following hepatectomy.

Among the four miRNAs of the exosomal miRNA classifier, exosomal miR-93-3p originated from tumor cells, while miR-132-5p, miR-320d, and miR-6087 from immunocytes. These four miRNAs can regulate tumor development, as previously reported [18,19,20]. Tissue miR-132-5p could impair the survival of hepatocellular cancer patients [21]. Tissue miR-6087 was previously outlined as a biomarker in bladder cancer and plays an important role in hepatocellular carcinoma development [20,22]. Although miR-6087 was discontinued in miRbase Version 22, the mature sequence of this miRNA was validated in our study and reported in other studies [22,23]. miRNA-93-3p could promote chemoresistance in triple-negative breast cancer and enhance clear cell renal cell carcinoma’s malignancy potential [19,24]. Elevated serum exosomal miR-320d was detected in metastatic CRC compared to nonmetastatic CRC (22) in a previous study, consistent with our results. Interestingly, miR-320d in tissue was reported to inhibit the development of several types of cancer, including CRC [18,25,26]. One plausible explanation for the high expression of miR-320d in exosomes is that malignant tumors may discharge suppressor genes into exosomes to increase the malignant phenotype [27,28].

Gene ontology (GO) enrichment analysis revealed that T cell activation was an enriched pathway for the 4 miRNA candidate target genes. Consistently, our results showed that expression of exosomal miR-132-5p, miR-320d, and miR-6087 was negatively related to Immunoscore in liver metastases. A previous study also reported that plasma exosomal miR-320d is upregulated in progressive disease compared with the partial response of immunotherapy in EGFR/ALK wild-type advanced nonsmall cell lung cancer [29]. These results imply that the miRNAs in our predictive model may influence the posthepatectomy prognosis by regulating the immune status of liver metastasis.

The necessity of adjuvant chemotherapy post CRLM resection remains controversial [30,31]. Our results showed that the high exosomal miRNA risk group, regardless of the CRS, had a better survival after adjuvant chemotherapy; hence, the 4-miRNA signature may help in stratifying patients that would benefit from adjuvant chemotherapy. Our study also had some limitations. The retrospective design, imbalanced clinical characteristics of different exosomal miRNA groups in Cohort 3, and the heterogeneity of the treatment between different centers weaken the generalizability of our results. Thus, the findings need to be interpreted cautiously. Additionally, although the GO analysis and Immunoscore results indicated a potential correlation between exosomal miRNAs and the tumor microenvironment, further investigation is needed for validation.

Taken together, the present study offers an insight into the predictive role of circulating exosomal miRNAs in CRLM and developed a predictive model for posthepatectomy prognosis and benefit from adjuvant chemotherapy. Further studies are needed to validate and extend the clinical application of this exosomal miRNA model.

## 5. Conclusions

We developed a circulating exosomal miRNA signature that can predict the prognosis and guide adjuvant chemotherapy decisions after liver metastasectomy in CRLM patients.

## Figures and Tables

**Table 1:** Clinicopathologic characteristics of patients with colorectal cancer liver metastases in three cohorts.

	Training Cohort			Internal Validation Cohort			External Validation Cohort		
	Low	High	*p*-Value	Low	High	*p*-Value	Low	High	*p*-Value
Age (years)									
≥70	8 (22.2)	5 (6.5)	**0.034** ^2^	5 (7.8)	6 (12.0)	0.531 ^2^	1 (8.3)	11 (7.1)	1.000 ^2^
<70	28 (77.8)	72 (93.5)		59 (92.2)	44 (88.0)		11 (91.7)	144 (92.9)	
Gender									
Male	26 (72.2)	46 (59.7)	0.199	43 (67.2)	33 (66.0)	0.894	9 (75.0)	109 (70.3)	1.000 ^2^
Female	10 (27.8)	31 (40.3)		21 (32.8)	17 (34.0)		3 (25.0)	46 (29.7)	
Tumor grade									
G3	5 (15.6)	21 (27.6)	0.183	14 (21.9)	8 (16.0)	0.430	1 (10.0)	13 (8.7)	1.000 ^2^
G1–2	27 (84.4)	55 (72.4)		50 (78.1)	42 (84.0)		9 (90.0)	136 (91.3)	
Primary tumor									
Rectal	17 (47.2)	29 (38.2)	0.362	21 (32.8)	13 (26.0)	0.430	3 (25.0)	62 (40.5)	0.368 ^2^
Colon	19 (52.8)	47 (61.8)		43 (67.2)	37 (74.0)		9 (75.0)	91 (59.5)	
T-stage ^1^									
Tis-2	3 (9.1)	10 (13.7)	0.750 ^2^	2 (3.2)	6 (12.5)	0.077 ^2^	1 (11.1)	12 (8.2)	0.553 ^2^
T3–4	30 (90.9)	63 (86.3)		60 (96.8)	42 (87.5)		8 (88.9)	135 (91.8)	
N-stage ^1^									
N0	18 (51.4)	31 (44.3)	0.489	25 (39.7)	20 (42.6)	0.762	5 (55.6)	51 (35.2)	0.287 ^2^
N1–2	17 (48.6)	39 (55.7)		38 (60.3)	27 (57.4)		4 (44.4)	94 (64.8)	
Interval to liver metastases ^3^ (months)									
>12	27 (75.0)	63 (82.9)	0.326	52 (81.3)	40 (80.0)	0.867	6 (50.0)	3 (19.4)	**0.023** ^2^
≤12	9 (25.0)	13 (17.1)		12 (18.8)	10 (20.0)		6 (50.0)	125 (80.6)	
Resection									
R0	28 (84.8)	49 (66.2)	**0.048**	51 (79.7)	40 (83.3)	0.625	12 (100.0)	138 (89.6)	0.609 ^2^
R1–2	5 (15.2)	25 (33.8)		13 (20.3)	8 (16.7)		0 (0.0)	16 (10.4)	
Ablation									
Yes	1 (2.8)	11 (14.3)	0.099 ^2^	10 (15.6)	7 (14.0)	0.809	1 (8.3)	10 (6.5)	0.571 ^2^
No	35 (97.2)	66 (85.7)		54 (84.4)	43 (86.0)		11 (91.7)	145 (93.5)	
Number of metastases per patient									
>1	19 (52.8)	47 (61.0)	0.406	45 (70.3)	30 (60.0)	0.249	5 (41.7)	103 (66.5)	0.116 ^2^
≤1	17 (47.2)	30 (39.0)		19 (29.7)	20 (40.0)		7 (58.3)	52 (33.5)	
Size of the max metastases (cm)									
>5	9 (25.0)	15 (19.5)	0.504	10 (15.6)	10 (20.0)	0.542	1 (8.3)	24 (15.5)	1.000 ^2^
≤5	27 (75.0)	62 (80.5)		54 (84.4)	40 (80.0)		11 (91.7)	131 (84.5)	
Preoperative CEA (ng/mL)									
>200	2 (5.6)	2 (2.6)	0.591 ^2^	3 (4.7)	3 (6.1)	1.000 ^2^	1 (8.3)	9 (5.8)	0.536 ^2^
≤200	34 (94.4)	75 (97.4)		61 (95.3)	46 (93.9)		11 (91.7)	146 (94.2)	
CRS									
0–2	24 (68.6)	40 (57.1)	0.258	36 (57.1)	27 (58.7)	0.871	7 (77.8)	78 (53.8)	0.188 ^2^
3–5	11 (31.4)	30 (42.9)		27 (42.9)	19 (41.3)		2 (22.2)	67 (46.2)	
Preoperative chemotherapy									
Yes	16 (44.4)	47 (61.0)	0.098	40 (62.5)	37 (74.0)	0.193	8 (66.7)	113 (72.9)	0.738 ^2^
No	20 (55.6)	30 (39.0)		24 (37.5)	13 (26.0)		4 (33.3)	42 (27.1)	
Postoperative chemotherapy									
Yes	27 (75.0)	48 (62.3)	0.184	51 (79.7)	31 (62.0)	**0.037**	9 (75.0)	89 (57.4)	0.363 ^2^
No	9 (25.0)	29 (37.7)		13 (20.3)	19 (38.0)		3 (25.0)	66 (42.6)	

CEA, carcinoembryonic antigen. CRS, clinical risk score. Data are presented as the number of cases followed by percentages in parentheses. The values in bold indicate that the difference between the correspondent characteristics groups was statistically significant (*p* < 0.05).^1^ According to the Union International Control Cancer (UICC) staging system (7th version) ^2^ Assessed by Fisher’s exact test ^3^ Interval from primary tumor resection to liver metastasis.

**Table 2:** Prognostic values for survival by univariate Cox analysis.

	Training Cohort				Internal Validation Cohort				External Validation Cohort			
	RFS		OS		RFS		OS		RFS		OS	
	HR (95% CI)	*p*-Value	HR (95% CI)	*p*-Value	HR (95% CI)	*p*-Value	HR (95% CI)	*p*-Value	HR (95% CI)	*p*-Value	HR (95% CI)	*p*-Value
Age												
≥70 vs. <70 years	0.82 (0.42–1.58)	0.553	0.98 (0.49–1.95)	0.944	1.00 (0.50–2.00)	0.994	1.46 (0.66–3.20)	0.348	0.53 (0.23–1.21)	0.131	1.04 (0.41–2.61)	0.932
Gender												
Male vs. female	0.82 (0.54–1.26)	0.368	0.83 (0.53–1.31)	0.429	1.09 (0.69–1.70)	0.719	1.15 (0.68–1.96)	0.598	0.93 (0.63–1.38)	0.723	0.91 (0.53–1.56)	0.739
Tumor grade												
G3 vs. G1–2	1.64 (1.02–2.64)	**0.043**	2.10 (1.27–3.48)	**0.004**	1.18 (0.69–2.00)	0.547	1.55 (0.87–2.76)	0.14	1.68 (0.94–3.02)	0.083	1.58 (0.71–3.50)	0.263
Primary tumor												
Rectal vs. colon	1.08 (0.71–1.63)	0.728	0.96 (0.62–1.50)	0.865	1.47 (0.95–2.29)	0.084	1.20 (0.72–2.01)	0.489	0.99 (0.69–1.44)	0.972	1.10 (0.65–1.85)	0.719
T-stage												
T3–4 vs. Tis-2	0.92 (0.49–1.73)	0.798	0.96 (0.49–1.86)	0.894	0.86 (0.39–1.85)	0.692	0.59 (0.25–1.37)	0.217	1.36 (0.66–2.79)	0.403	1.47 (0.53–4.07)	0.460
N-stage												
N1–2 vs. N0	1.23 (0.80–1.89)	0.35	1.95 (1.22–3.13)	**0.006**	1.32 (0.84–2.07)	0.224	1.33 (0.79–2.25)	0.287	1.16 (0.78–1.72)	0.465	1.15 (0.65–2.02)	0.636
Interval to liver metastases ^1^												
>12 vs. ≤12 months	1.58 (0.92–2.72)	0.099	1.42 (0.78–2.58)	0.247	0.70 (0.42–1.17)	0.171	0.77 (0.42–1.39)	0.385	1.30 (0.84–2.01)	0.238	0.93 (0.52–1.68)	0.818
Resection												
R1–2 vs. R0	2.14 (1.36–3.37)	**0.001**	3.60 (2.21–5.85)	<**0.001**	2.30 (1.38–3.82)	**0.001**	1.88 (1.06–3.32)	**0.031**	1.53 (0.88–2.68)	0.135	2.11 (0.99–4.50)	0.053
Ablation												
Yes vs. no	2.01 (1.07–3.79)	0.03	1.95 (1.02–3.70)	**0.042**	1.77 (1.01–3.09)	**0.045**	1.62 (0.88–2.99)	0.121	3.06 (1.62–5.79)	**0.001**	0.5(0.13–2.20)	0.385
Number of metastases per patient												
>1 vs. ≤1	1.97 (1.28–3.03)	0.002	1.97 (1.24–3.14)	**0.004**	1.68 (1.07–2.63)	**0.024**	1.78 (1.02–3.10)	**0.042**	1.95 (1.31–2.90)	**0.001**	1.00 (0.59–1.69)	0.994
Size of the max metastases												
>5 vs. ≤5 cm	0.92 (0.55–1.54)	0.749	1.06 (0.62–1.81)	0.842	1.32 (0.77–2.27)	0.312	2.08 (1.14–3.78)	**0.016**	1.21 (0.75–1.93)	0.436	1.89 (1.06–3.36)	**0.030**
Preoperative CEA												
>200 vs. ≤200 ng/mL	1.22 (0.38–3.87)	0.736	1.97 (0.62–6.26)	0.253	3.03 (1.29–7.10)	**0.011**	3.22 (1.27–8.14)	**0.013**	1.72 (0.84–3.55)	0.138	1.35 (0.54–3.39)	0.519
CRS												
	1.27 (1.03–1.58)	**0.027**	1.42 (1.13–1.79)	**0.003**	1.28 (1.03–1.59)	**0.029**	1.48 (1.12–1.95)	**0.005**	1.31 (1.07–1.60)	**0.008**	1.09 (0.83–1.44)	0.528
Risk score												
High vs. low	3.11 (1.88–5.15)	<**0.001**	3.65 (2.06–6.46)	<0.001	1.67 (1.10–2.54)	**0.016**	2.39 (1.45–3.95)	**0.001**	3.33 (1.35–8.21)	**0.009**	4.61 (1.11–19.03)	**0.035**

CEA, carcinoembryonic antigen. CRS, clinical risk score. Bold indicates *p* < 0.05. ^1^ Interval from primary tumor resection to liver metastasis.

## Data Availability

The authenticity of this article was validated by uploading the key raw data onto the Research Data Deposit public platform (www.researchdata.org.cn (accessed on 18 August 2021)).

## Supplementary Materials

The following are available online at https://www.mdpi.com/article/10.3390/cancers13174258/s1, Figure S1: Flowchart of the exosomal miRNA model construction and validation, Figure S2: Isolated exosome characterization. Figure S3: The expressions of differential exosomal miRNAs were compared between 10 nonmetastatic CRC and 20 CRLM plasma samples by qRT-PCR. Figure S4: A merged risk score was constructed based on the circulating exosomal miRNA risk score and the CRS system. Figure S5: The Sanger sequencing of the products of the qPCR array for the 4 model-related miRNAs. Table S1: Prognostic values for survival by multivariate Cox analysis.
