# Supplementary material for: A Prehepatectomy Circulating Exosomal microRNA Signature Predicts the Prognosis and Adjuvant Chemotherapeutic Benefits in Colorectal Liver Metastasis
Source: Cancers (Basel). 2021 Aug 24;13(17):4258. doi: 10.3390/cancers13174258 (PMC8428239; doi:10.3390/cancers13174258)
Supplement: Supplementary file 1 [file cancers-13-04258-s001.zip › cancers-1270216-supplementary.pdf]

Supplemental Tables:

Table S1: Prognostic values for survival by multivariate Cox analysis.

		RFS		OS	
		HR (95%CI)	P-value	HR (95%CI)	P-value
**Training cohort**					
Tumor grade					
	G3 *vs.* G1-2	1.07(0.63-1.80)	0.804	1.56(0.91-2.70)	0.108
Resection					
	R1-2 *vs.* R0	1.22(0.71-2.08)	0.474	2.69(1.51-4.79)	**0.001**
Ablation					
	Yes *vs.* no	2.13(1.04-5.39)	**0.040**	0.99(0.48-2.05)	0.984
CRS					
		1.24(0.98-1.56)	0.069	1.31(1.02-1.70)	**0.035**
Risk score					
	High *vs.* low	2.43(1.40-4.21)	**0.002**	2.70(1.44-5.06)	**0.002**
**Internal validation cohort**					
Resection					
	R1-2 *vs.* R0	2.48(1.38-4.47)	**0.003**	1.72(0.92-3.22)	0.091
Ablation					
	Yes *vs.* no)	2.16(1.16-4.03)	**0.015**	-	-
CRS					
		1.07(0.83-1.38)	0.594	1.43(1.06-1.92)	**0.018**
Risk score					
	High *vs.* low	1.82(1.17-2.82)	**0.008**	2.51(1.48-4.25)	**0.001**
**External validation cohort**					
Ablation					
	Yes *vs.* no	3.00(1.53-5.88)	**0.001**	-	-
Size of the max metastases					
	> 5 *vs.* ≤ 5 cm	-	-	1.75(0.98-3.12)	0.058
CRS					
		1.31(1.06-1.62)	**0.011**	-	-
Risk score					
	High *vs.* low	2.74(1.00-7.50)	**0.050**	4.26(1.03-17.63)	**0.046**

CRS clinical risk score. Bold indicate $P < 0.05$.

Supplemental Figures:

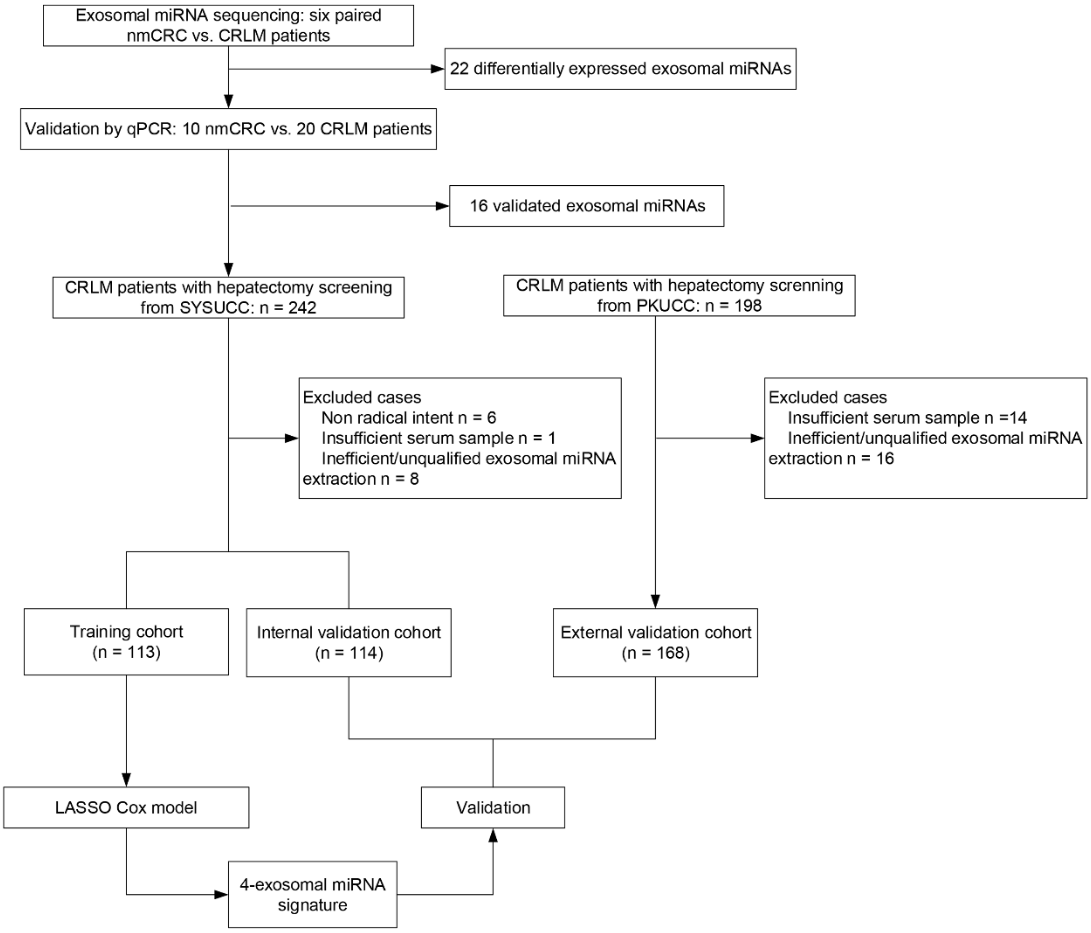

Figure S1. Flow chart of the exosomal miRNA model construction and validation.

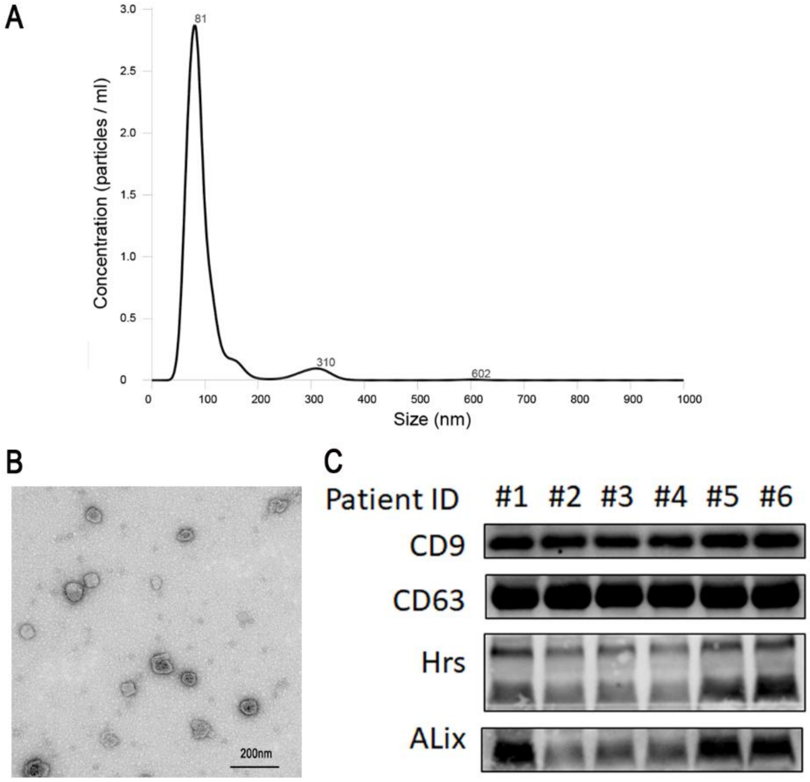

Figure S2. Isolated exosome characterization. The isolated exosomes were identified by NanoSight NS300 (A), electron microscope (B) and exosome markers CD9, CD63, HRS and Alix based on western blot (C).

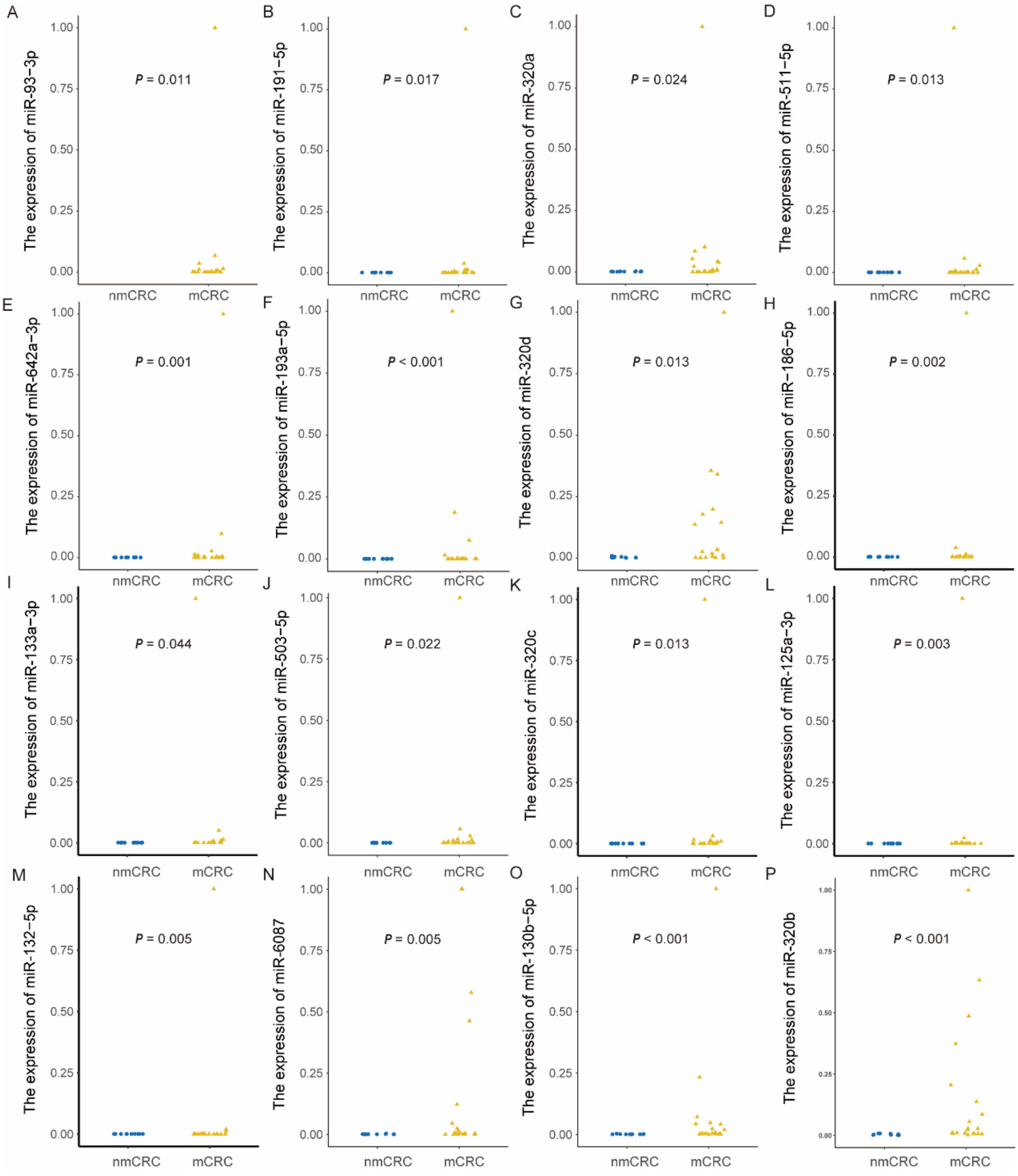

Figure S3. The expressions of differential exosomal miRNAs were compared between 10 non-metastatic CRC and 20 CRLM plasma samples qRT-PCR. Data was analyzed by t-test, only differential exosomal miRNAs with statistical significance were presented.

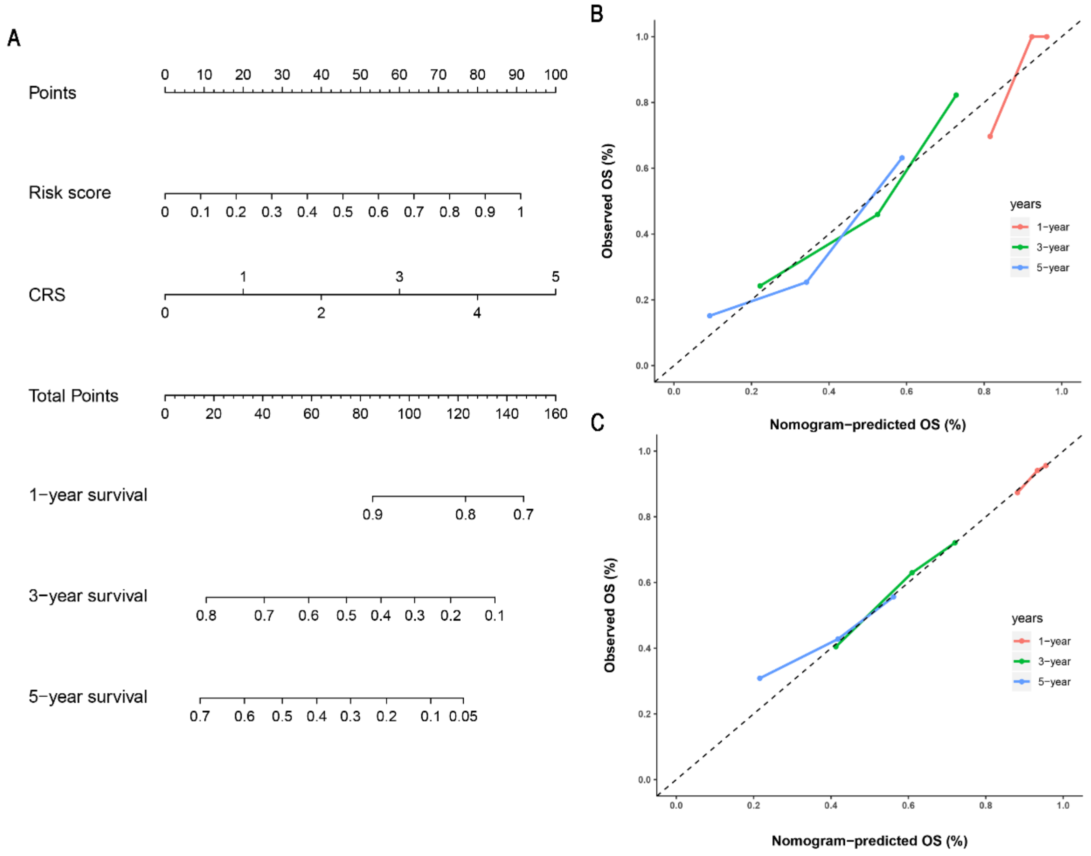

Figure S4. A merged risk score was constructed based on the circulating exosomal miRNA risk score and CRS system. A. A merged score for survival prediction was constructed by nomogram based on the circulating exosomal miRNA risk score and CRS system in the training cohort. B-C. The calibration curves of the nomogram were drawn in terms of the agreement between the predicted and observed survival in the training cohort (B) and combined validation cohorts (C).

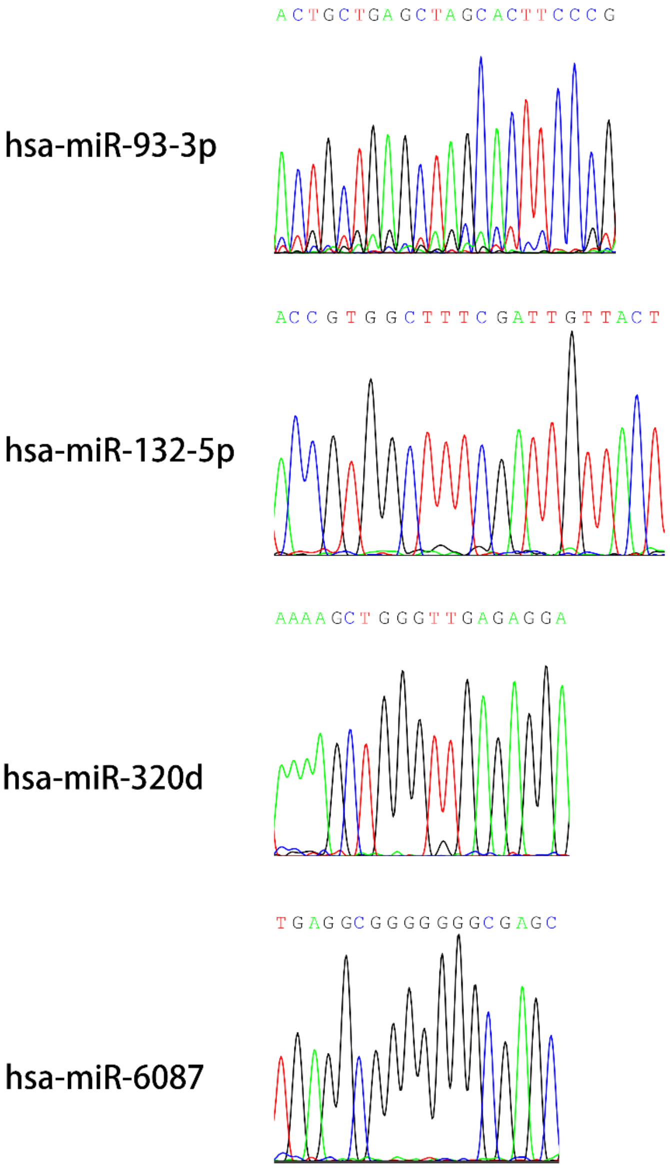

Figure S5. The sanger sequencing of the products of the qPCR array for the 4 model-related miRNAs.
